# A novel insulin delivery system by β cells encapsulated in microcapsules

**DOI:** 10.3389/fchem.2022.1104979

**Published:** 2023-01-05

**Authors:** Zongjie Luo, Yutong Dong, Mengyu Yu, Xiao Fu, Yudong Qiu, Xitai Sun, Xuehui Chu

**Affiliations:** Department of General Surgery, Nanjing Drum Tower Hospital Clinical College of Traditional Chinese and Western Medicine, Nanjing University of Chinese Medicine, Nanjing, China

**Keywords:** β cells, insulin delivery system, microfluidic, microcapsules, diabetes

## Abstract

**Introduction:** Diabetes is a growing epidemic worldwide and requires effective clinical therapies. In recent years, β-cell transplantation has emerged as a promising treatment for diabetes, and an encapsulation approach has been proposed to ameliorate this treatment.

**Methods:** Microfluidic technology had been used to generate microcapsules using a porous sodium alginate shell and a core containing β cells. The microcapsules were transplanted into diabetic mice and the therapeutic effect was measured.

**Results:** Porous hydrogel shell allows exchange of small molecules of nutrients while protecting beta cells from immune rejection, while the core ensures high activity of the encapsulated cells. The glucose control effect of the microcapsules were more durable and better than conventional methods.

**Discussion:** We believe that this system, which is composed of biocompatible porous hydrogel shell and enables highly activity of encapsulated β cells, can enhance therapeutic efficacy and has promising clinical applications.

## 1 Introduction

Diabetes is a serious endocrine system disease worldwide, causing secondary damage to several organs of the body (The Prevention of Diabetes Mellitus, 2021; Cloete, 2022). Both type 1 diabetes and late-onset type 2 diabetes manifest as absolute insulin deficiency (DiMeglio et al., 2018). Recently, several treatments for this condition have been used, such as oral medications, insulin injections, and islet transplants (Shahjalal et al., 2018; Yoshihara et al., 2020; Cloete, 2022). Oral medications and insulin injections increase the burden on diabetic patients and require frequent medication. Islet transplantation has been performed for many years and has been proven to be effective, but the duration and survival rate of islet transplantation are greatly reduced after a long period due to the attack of immune cells (Aguayo-Mazzucato and Bonner-Weir, 2018; Bourgeois et al., 2021; Brusko et al., 2021). Therefore, biocompatible hydrogels were applied to protect β cells from the immune cells (Espona-Noguera et al., 2019; Reys et al., 2022). Three main methods of cell encapsulation have been put forward: hydrogel encapsulation, utilizing porous devices, and thin polymer coating (van Bochove and Grijpma, 2019; Khayambashi et al., 2021; Peng et al., 2021). Despite some successes, these methods lead to either suboptimal viability of the encapsulated cells or a fibrotic response. In addition, they lack the ability to exchange substances, making it difficult for the encapsulated cells to survive in the long term. Therefore, there is an urgent need for a novel drug delivery method that enables both long-term cell survival *in vivo* and a good exchange of substances (Wong et al., 2018).

In our research, we fabricated a novel microcapsule that encapsulates β cells using microfluidic technology to treat diabetes. Microcapsules are core–shell particles with a diameter of 1–1,000 μm made using natural or synthetic polymeric materials (Zhao et al., 2019; Huang et al., 2022). Various functional microcapsules have been developed by different technological applications, such as microfluidics, spray drying, and interfacial polymerization (Chen et al., 2019). Among them, microfluidics is a well-known strategy for rapidly preparing microcapsules with the desired morphology (Kim et al., 2022). The obtained microcapsules can effectively encapsulate the cells, but the cell viability in them is usually not adequate because the hydrogel shell reduces cell viability and intercellular communication and the compact shell restricts the internal cells from accessing external nutrients and limits insulin exiting the enclosure (Khanmohammadi et al., 2020; Liu et al., 2021; Kim et al., 2022). Therefore, the creation of a microcapsule that encapsulates highly active cells for diabetes treatment is imminent.

Here, we used a facile microfluidic electrospray device with double coaxial capillaries to prepare the microcapsules. The microcapsule shell is made of a mixture of sodium alginate (ALG) and cellulose nanocrystal (CNC). The shell with CNC endows the system with enough mechanical strength and a unique porous structure, which facilitates the exchange of substances between the cells and the external environment. The shell also avoids immune damage during treatment. The core is composed of carboxymethyl cellulose (CMC) solution and β cells. The liquid core of the microcapsules provides the encapsulated β cells with a 3D culture environment, while the shell protects these cells from immune cells after transplantation. Moreover, the encapsulated β cells are exposed to body fluid through the pores of the microcapsules, and insulin is released according to the current blood glucose status. Insulin can be released directly into the body through the pores. All these features suggest that β cells encapsulated in porous microcapsules show unique potential in diabetes therapy, which makes them a promising candidate for further clinical studies.

## 2 Materials and methods

### 2.1 Materials

ALG, CMC, and CaCl_2_ were bought from Sigma. CNC was obtained from Beike 2D Materials. Cell counting kit-8 and calcein/PI were purchased from KeyGene. The insulin-producing β-cell line was obtained from ATCC. Seven-week-old female C57BL/6 mice weighing 20–22 g were acquired from the Model Animal Research Center of Nanjing University. All animal treatments were performed in strict compliance with the guidelines approved by the Animal Ethics Committee of Nanjing Drum Tower Hospital.

### 2.2 Design of the microfluidic device

The microfluidic electrospray tip device was set by assembling two circular capillaries coaxially. The diameters of the inner and outer capillaries, respectively, were 100 and 300 μm. The inner capillary was inserted coaxially into the outer capillary. The attachment points of the device were then sealed with clear glue.

### 2.3 Fabrication and characterization of porous microcapsules

In a typical experiment, two syringe pumps were used to push the shell fluid of 1.5% (w/v) ALG with .5% (w/v) CNC and the core fluid of 2% (w/v) CMC through the concentric outer (300 μm) and inner (100 μm) lumens in the coaxial needle. A 5 KV electrostatic potential was applied to the microfluidic device to generate droplets. Then, the droplets were collected in 2% (w/v) CaCl_2_ solution. The microcapsules were generated by rapid crosslinking between Ca^2+^ and ALG.

### 2.4 Biocompatibility evaluation

The viability of β cells was measured by cell counting kit-8 (CCK-8) assay. The prepared porous microcapsules with uniform dimensions were selected under the microscope. The β cells were planted in a 96-well plate. β cells co-cultured with microcapsules were regarded as the experimental group, and β cells cultured alone were regarded as the control group. After 72 h, the CCK-8 experiment was conducted.

### 2.5 Cell encapsulations and culture

β cells (2 × 10^6^ cells/ml) in CMC (2%, w/v) were used as the core fluid, and 1.5% (w/v) ALG and .5% (w/v) CNC were used as the shell fluid. After encapsulation, the microcapsules were washed three times with a culture medium and preserved in a culture medium.

### 2.6 Animal experiment

Female C57 mice were intraperitoneally injected with streptozotocin (STZ) (150 mg/kg) to establish a diabetic model. Blood glucose levels were measured every day after the administration of STZ. The level of blood glucose greater than 16.7 mmol/L showed the successful establishment of the diabetic model. Diabetic mice transplanted with microcapsules containing β cells were designated as the β-cell-Mi group. Mice that received dispersed β cells were designated as the β-cell group. Sham-operated animals were designated as the control group. For the implantation surgery, diabetic mice were injected intraperitoneally with sodium pentobarbital 60 mg/kg. A total of 300 microcapsules were placed into the mesenteric pouch, which was subsequently placed back into the mouse’s abdominal cavity, and the mouse skin was carefully sutured. The mice’s body weight and blood glucose levels were tracked and recorded every other day. A week after transplantation, the mice were injected intraperitoneally with 15% glucose after fasting for 12 h. The blood glucose levels were then measured at 0, 15, 30, 60, 90, and 120 min. The mice were executed after 21 days of treatment. Blood count and liver function were measured. The heart, liver, spleen, lungs, and kidneys were fixed for 24 h. The cells were then stained with hematoxylin and eosin (H&E) and were observed under the microscope and photographed.

## 3 Results and discussion

### 3.1 Synthesis of the porous microcapsules

**FIGURE 1:**
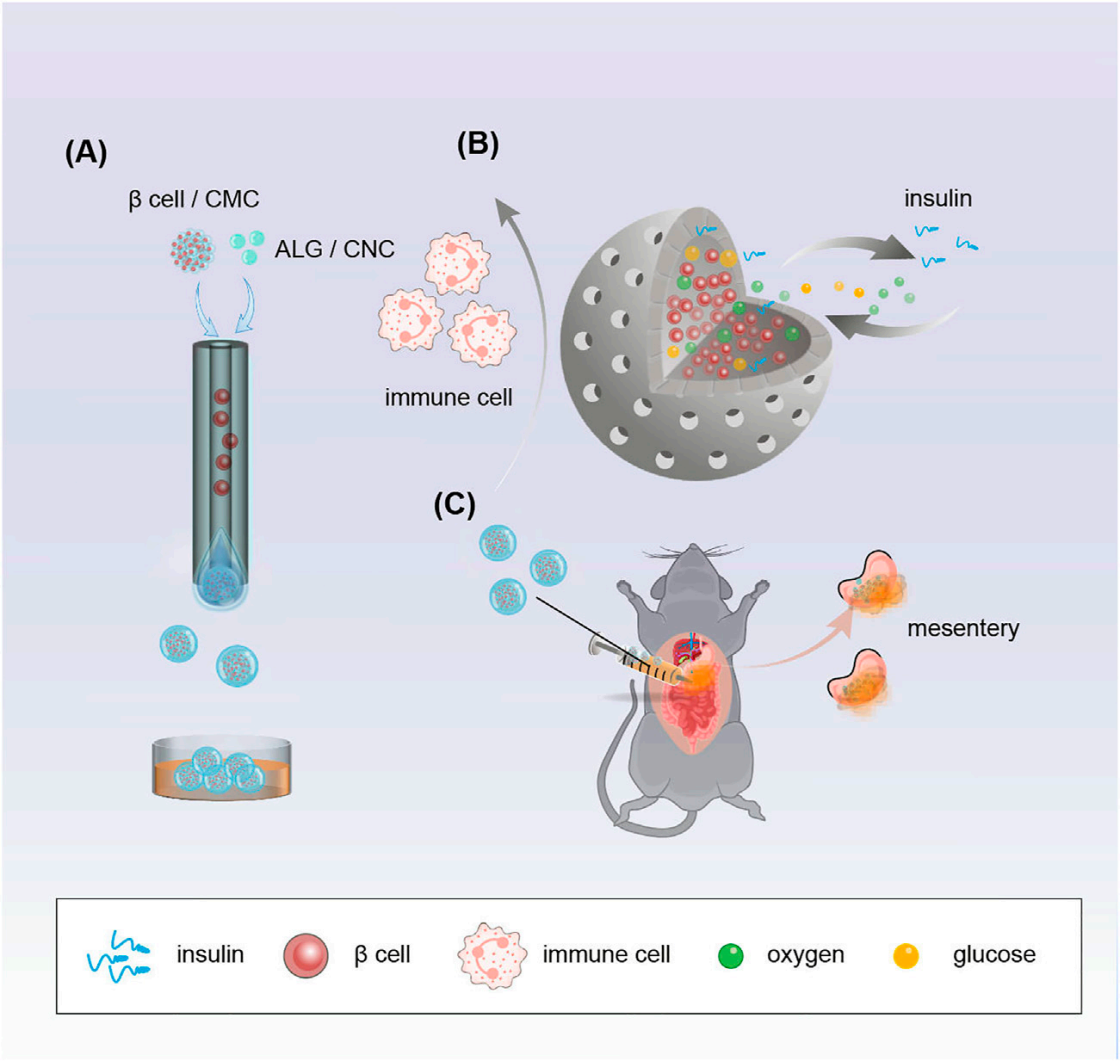
Schematic demonstrating the fabrication of porous microcapsules encapsulating β cells for diabetes treatment after mesentery transplantation in diabetic mice. **(A)** Porous microcapsules fabricated *via* the microfluidic electrospray system. **(B)** β-cell-encapsulated microcapsules isolate immune cells, release insulin, and exchange oxygen and glucose. **(C)** β-cell-encapsulated porous microcapsules were applied to treat diabetes in diabetic mice after mesentery transplantation.

**FIGURE 2:**
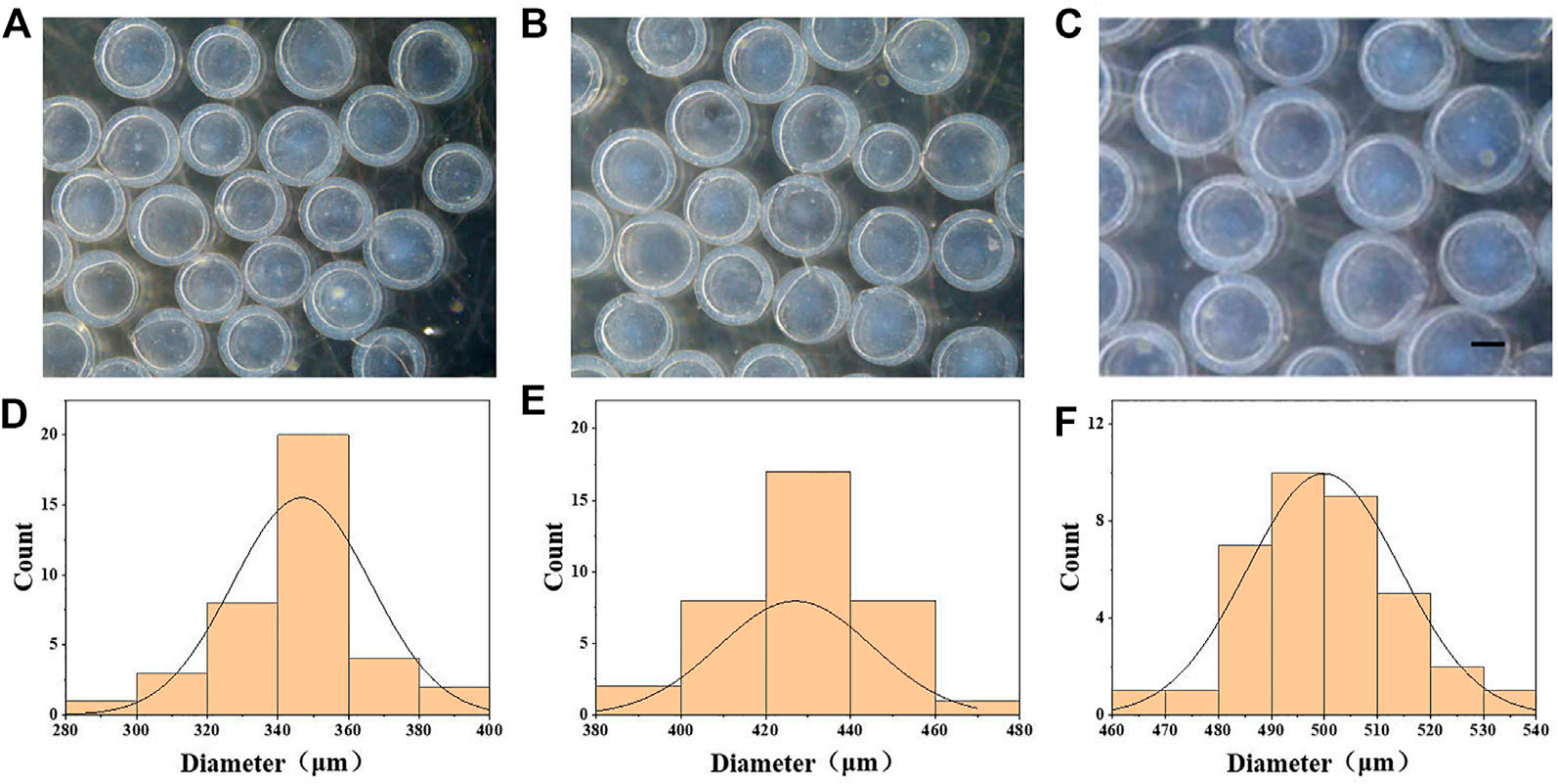
Generation of CMC–ALG microcapsules *via* microfluidic electrospray technology. **(A–C)** Bright-field microscopic images of the CMC–ALG core–shell microcapsules with different voltages. Scale bar represents 200 μm. **(D–F)** Diameter distribution of microparticles under different voltages. **(A)** 6 kv, **(B)** 7 kv, and **(C)** 8 kv.

In a typical experiment, β-cell-encapsulated microcapsules were achieved as shown in Figure 1. The outer phase of the device consists of 1.5% ALG and .5% CNC. The inner phase consists of β cells in a culture medium with 2% CMC. The droplets were collected in CaCl_2_ solution. ALG was instantly solidified to form microcapsules by the fast diffusion of Ca^2+^. Cell-encapsulated microcapsules were rapidly collected and washed with the culture medium. Then, the microcapsules were photographed using the optical microscope. The size and structure of the microcapsules were controlled by pulsed electric field voltage, collection distance, the consistency of the biomass solution, and flow rates of internal and external phases (Figures 2A–F). The size of the microcapsules decreases with the increasing voltage, decreasing collecting distance, and decreasing biomass solution concentration. The decreasing flow rate of the internal phase led to decreasing diameters of cores, increasing shell thickness, and a slightly decreasing diameter of the entire capsule. Furthermore, the decreasing flow rate of the outer phase led to the diminishing diameter of the microcapsule and the shell thickness, while increasing the core diameter.

### 3.2 Preparation of the porous microcapsules and *in vitro* culture

**FIGURE 3:**
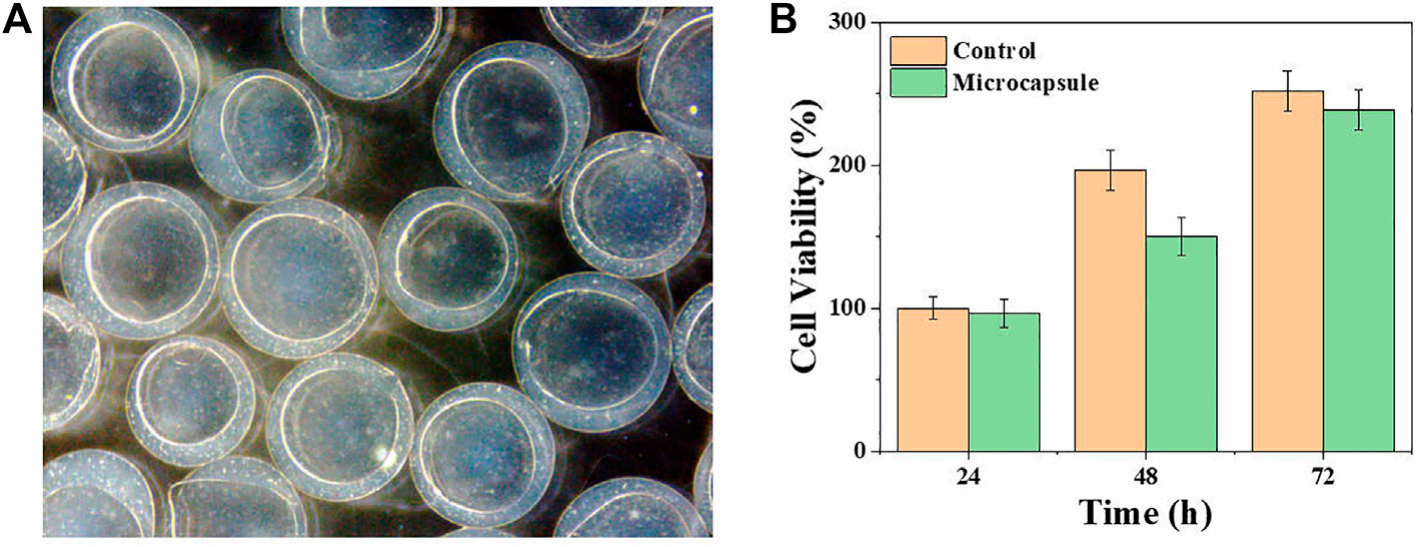
Viability and proliferation of β cells encapsulated in porous microcapsules. **(A)** Microscopic images of β cells after microencapsulation. **(B)** CCK-8 results of β cells of the control and microcapsule groups.

The biocompatibility of microcapsules was tested to investigate their biomedical application potential. The viability of the β cells was measured when cultured with or without the microcapsules. Specifically, β cells were co-cultured with (regarded as the microcapsule group) or without (regarded as the control group) the microcapsules in 96-well plates for 3 days. The OD values were detected, depending on the amount of cells per well, and the number of live cells were counted by CCK8 to analyze the growth status (Figure 3B). The results showed that the cell viability between the two groups was not noticeably different during 3 days of cultivation. These results demonstrated great biocompatibility of the microcapsules. Then, β cells were wrapped inside the microcapsules and kept sterile throughout. The cells were incubated in an incubator at a constant temperature, observed daily using a microscope, and photographed. In Figure 3A, it is obvious that the cells inside the microcapsules proliferate with time.

### 3.3 Animal experiment

**FIGURE 4:**
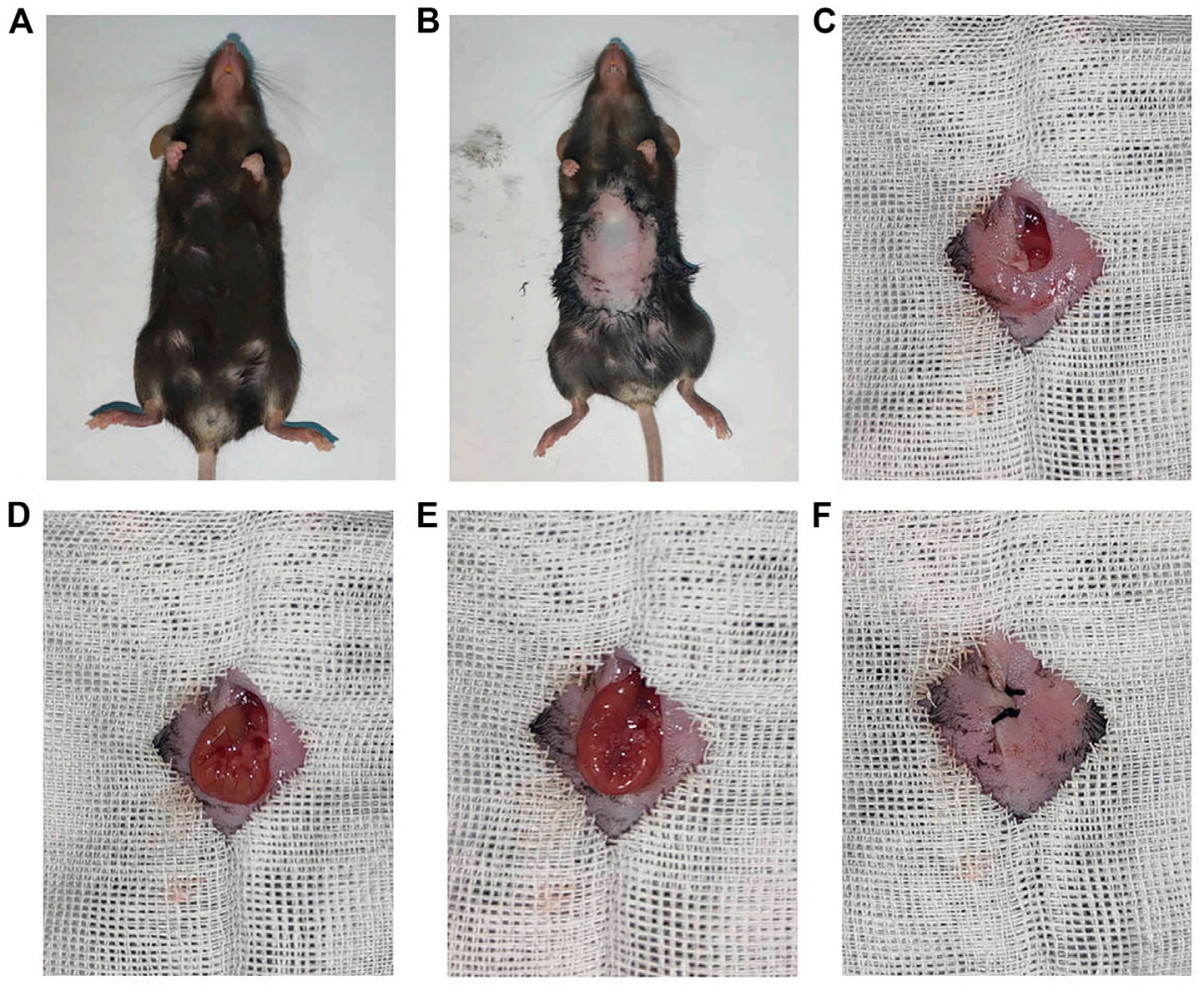
Procedure of β-cell-encapsulated microcapsules transplanted to the mesentery. **(A,B)** General pictures of a mouse. **(C–F)** Intraperitoneal delivery of microcapsules to the mouse with diabetes.

**FIGURE 5:**
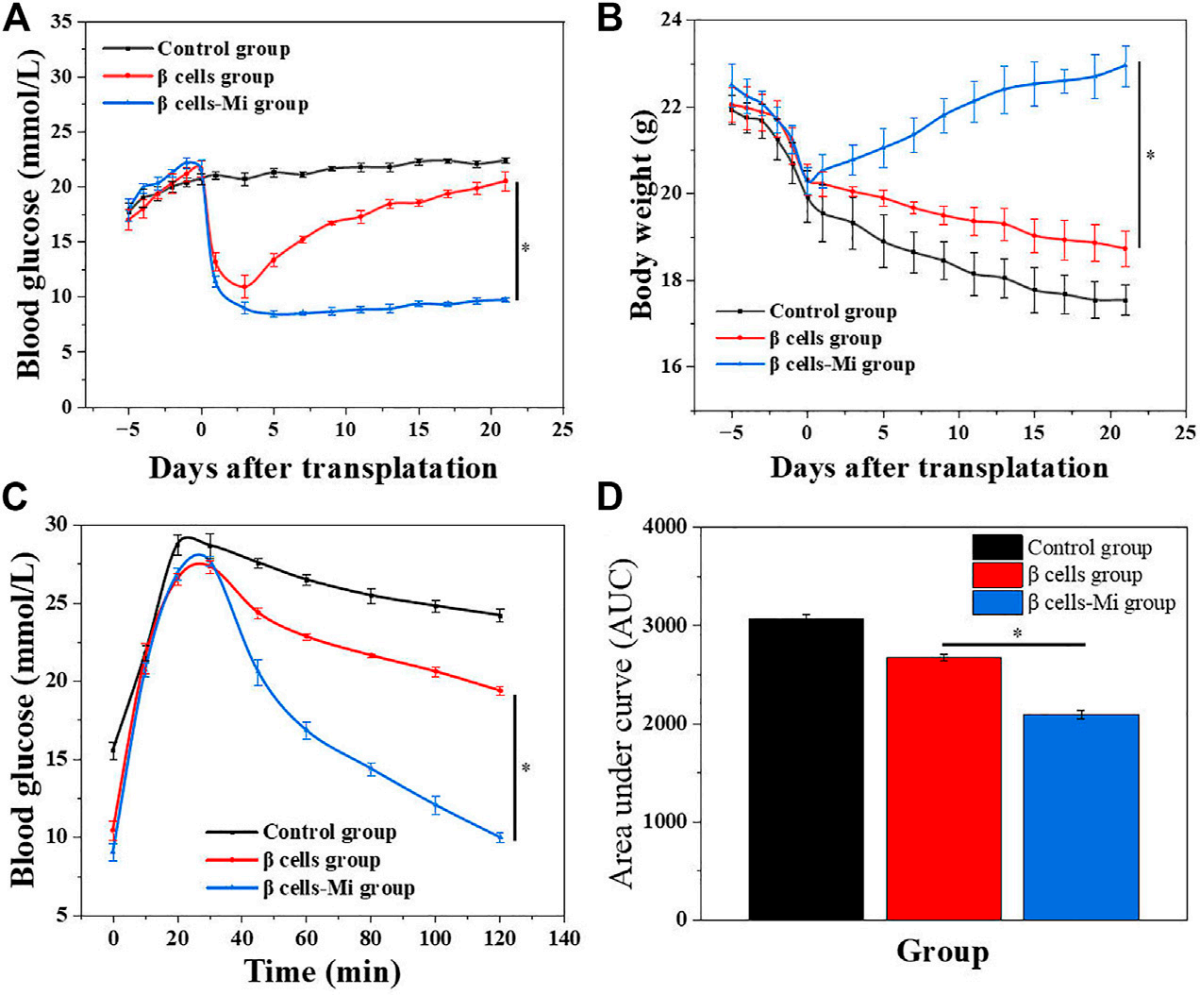
*In vivo* anti-diabetic efficiency of the β-cell-encapsulated microcapsules. **(A,B)** Blood glucose levels and body weights after transplantation. **(C)** Glucose tolerance tests in diabetic mice in different groups 2 h post-administration. **(D)** Responsiveness was calculated based on the area under the curve (AUC) at 120 min.

**FIGURE 6:**
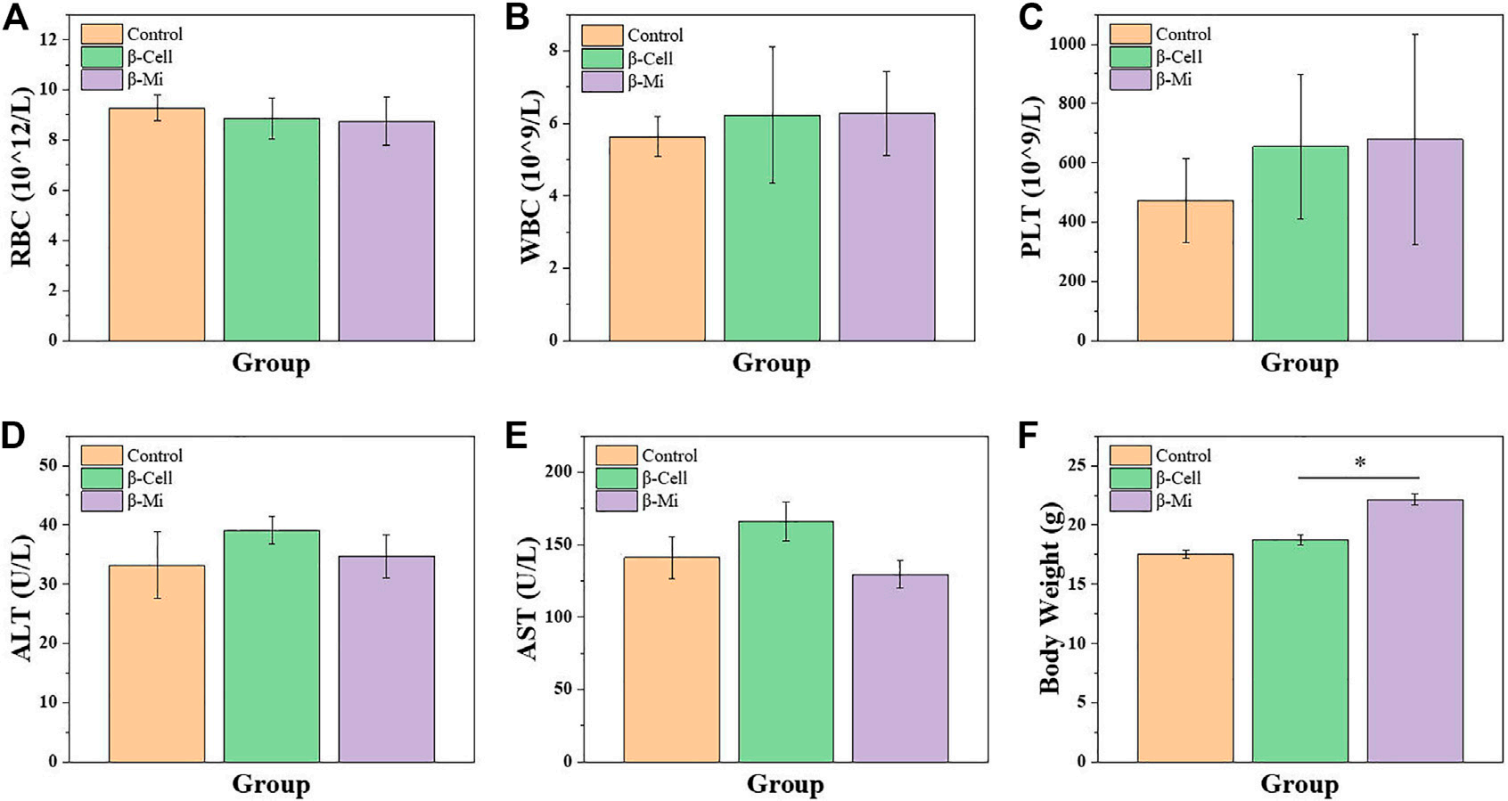
Biosafety analysis of β-cell-Mi therapy *in vivo*. **(A–E)** Hematology results of mice after treatments. **(F)** Body weight of mice before sacrifice.

**FIGURE 7:**
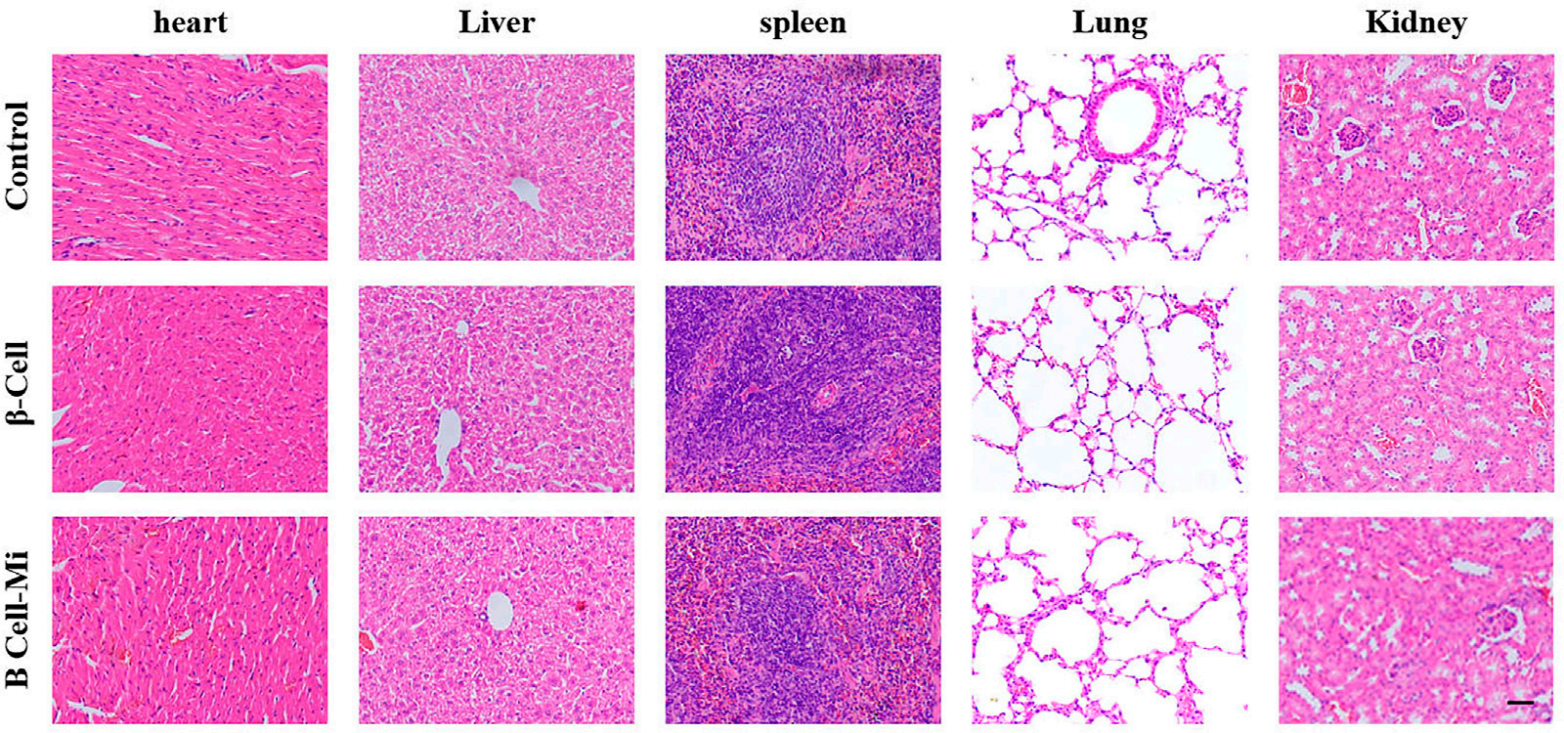
H&E staining of the main organs. Scale bar represents 200 μm.

In the end, these porous microcapsules encapsulating β cells were transplanted into diabetic mice to examine the therapeutic effects of treating diabetes. First, C57 mice were injected intraperitoneally with streptozotocin to establish a diabetic mouse model. The mice were divided randomly into the β-cell-Mi group, β-cell group, and control group. The process of transplanting the microcapsules is shown in Figure 4. Then, the blood glucose levels and body weight of the diabetic mice were recorded every other day (Figures 5A, B). The mice in the β-cell-Mi group returned to normal blood glucose levels and showed a slight increase in their body weight. The blood glucose level of the β-cell group also recovered. After 5 days, the blood glucose level gradually increased and the weight of the mice began to reduce. In addition, a mouse died on the 10th day. The mice in the control group maintained their diabetic status with a high blood glucose level and loss of weight. These measurements suggested that the direct transplantation of β cells may lead to a serious immune response. In contrast, the β cells encapsulated in microcapsules were protected by the shell and showed a long-term therapeutic effect. The intraperitoneal glucose tolerance test was performed on the seventh day (Figure 5C). The blood glucose levels of mice in the β-cell-Mi group returned to normal levels within 2 h. The blood glucose levels of mice in the β-cell group decreased significantly compared to those in the control group (Figure 5D). However, the normal blood glucose level was not decreased. These results demonstrated that the mice treated with β-cell microcapsules had great tolerance to glucose, which should be attributed to the continuous insulin release from the microcapsules. Finally, the mice were executed after 21 days of treatment. The complete blood count and liver function examination were in normal scope (Figures 6A–E). The organs were removed for H&E staining and were photographed (Figure 7). HE staining showed no obvious damage to the organs of all groups of mice, demonstrating the excellent biocompatibility of the microcapsules.

## 4 Conclusion

In summary, we presented novel porous microcapsules using microfluidic electrospray technology to encapsulate β cells to treat diabetes. In the preparation process, microcapsules containing a porous hydrogel shell and a β-cell core were prepared using a coaxial capillary device. The shell of porous hydrogel was made of a mixture of ALG and CNC, and the internal core was made of CMC solution with β cells. Driven by an external electric field, the co-current was disintegrated into droplets and falls into CaCl_2_ solution. Since gelation of ALG occurs rapidly, the porous shell of the microcapsule was immediately obtained, which protects the packaged β cells from immune attack after transplantation and allows the exchange of small molecules required for β-cell survival. In addition, the insulin secreted by β cells dispersed through the shell efficiently. The internal fluid core provides β cells a three-dimensional culture environment that preserves perfect cell function. Using a microfluidic electrospray method, microcapsules with tunable morphology allow for the controlled release of insulin. The released insulin from the microcapsules showed satisfactory antidiabetic function. Specifically, the blood glucose of mice in the microcapsule group was significantly lower than that in the β-cell transplantation group. Meanwhile, the body weight of mice in the microcapsule group was significantly regained to the normal level. These characteristics suggest that microcapsules encapsulating β cells in the porous form are effective in the treatment of diabetes. Therefore, we believe that this approach will have the potential to replace conventional β-cell transplantation in clinical use.

## Data Availability

The raw data supporting the conclusion of this article will be made available by the authors, without undue reservation.
